# Electron transport through nanoscale multilayer graphene and hexagonal boron nitride junctions

**DOI:** 10.3762/bjnano.16.147

**Published:** 2025-11-24

**Authors:** Aleksandar Staykov, Takaya Fujisaki

**Affiliations:** 1 International Institute for Carbon Neutral Energy Research (WPI-I2CNER), Kyushu University, Japan; 2 Faculty of Materials for Energy, Shimane University, Japan

**Keywords:** NEGF, DFT, nano-junction

## Abstract

In this study, we employ the non-equilibrium Green’s function (NEGF) method combined with density functional theory (DFT) to compare electron transport through several layers of nanoscale graphene and hexagonal boron nitride (h-BN). Calculations were performed for one to six layers, corresponding to thicknesses of 0.5–3.0 nm, respectively. Electron transport was computed perpendicular to the layers in the stacking direction. We compared the decay of the current with the number of layers and evaluated the ability of h-BN to filter currents as a material coating. To investigate the effect of disorder, we included two major defects in the graphene lattice, namely, nitrogen doping and Stone–Wales defects. Nitrogen doping transforms graphene from a zero-bandgap semiconductor to a metal, while Stone–Wales defects open the bandgap. For h-BN, we considered Stone–Wales defects. A detailed comparison of electron transport through five materials, that is, multilayer nanoscale graphene, N-doped multilayer nanoscale graphene, Stone–Wales-defective multilayer nanoscale graphene, h-BN, and Stone–Wales-defective h-BN allowed us to understand the currents at the nanoscale and the chemical and structural control over the electron transport. The slopes of the current decay with thickness enabled us to extrapolate trends for electron transport in thicker multilayer carbon and h-BN materials.

## Introduction

Multilayer graphene nanomaterials exhibit interesting electronic properties and find application in battery electrode materials [1], electrocatalysis [2], nanoscale electronics and electronic components [3], thermoelectric devices [4], and transparent films [5]. Graphene is a two-dimensional zero-bandgap semiconductor with excellent bulk conductivity. Its in-plane electron transport strongly depends on lattice order, lattice defects, and three-dimensional lattice curvature [6]. The in-plane electron transport is a result of the high charge carrier mobility in the two-dimensional π-conjugated electron system. Deviation from the perfect hexagonal lattice by the insertion of heteroatoms, structural defects, or local disorder, creates electron trapping sites and hinders the electron mobility [7]. In addition, zigzag and armchair edges in graphene nanoribbons will open the bandgap and create trapping sites. Thus, small graphene nanoribbons will show higher electrical resistance compared to larger nanoribbons and infinite two-dimensional sheets. The in-plane and the inter-plane electron transport in nanoscale graphene are strongly related to the overlap integral between the 2p*_z_* atomic orbitals (AOs) at adjacent carbon sites given in Equation 1. The overlap integral is a function of the distance and angle between the two 2p*_z_* AOs. In-plane overlap has π-character (strong overlap) while inter-plane overlap has σ-character (weak overlap) [8]. The distance between carbon atoms plays a primary role for the value of the overlap integral, leading to large π-overlaps in-plane and small inter-plane σ-overlaps.


[1] $$S=\int {\text{p}}_{z}{\text{p}}_{z}\text{d}V=-\cos\varphi \int {\text{p}}_{\sigma }{\text{p}}_{\sigma }\text{d}V+\sin\varphi \int {\text{p}}_{\pi }{\text{p}}_{\pi }\text{d}V.$$

The research in electron transport in nanoscale graphene has focused on in-plane conductivity, and only very few studies have reported data on the electron transport properties between the adjacent planes [9–13]. Defects in graphene can alter its electronic states and lead to deviations from its intrinsic properties. We look at two common defects in two-dimensional graphene, that is, N-substitution of carbon atoms and Stone–Wales defects [7]. Nitrogen substitution leads to three distinct states, namely, pyridinic, pyrrolic, and graphitic [14]. While pyridinic and pyrrolic nitrogen involves graphene edges, the graphitic nitrogen can be seen as a bulk defect. In this work, we deal with periodic graphene models, and we consider only graphitic nitrogen substitutions. The graphitic nitrogen substitution in graphene changes its properties from a zero-bandgap semiconductor to metallic character and decreases its work function [7]. Stone–Wales defects are results of C–C bond rotation in the graphene plane, which isomerizes a 6–6 ring structure to a 7–5 ring structure. Stone–Wales defects are commonly observed in multilayer graphene, graphite, graphene oxide, and their occurrence is temperature-dependent [15–16].

Hexagonal boron nitride (h-BN) is a material that shares many structural and electronic properties with graphite. It is isoelectronic to graphene with N contributing two electrons to the π-conjugated bond and B contributing zero electrons, as opposed to graphene, where each C atom contributes one electron. The h-BN structure is composed of polar bonds with N acting as electron donor and B acting as electron acceptor. Unlike graphene and graphite, h-BN shows high resistivity and is a large-bandgap semiconductor [17]. h-BN has a wide range of applications due to its unique properties [18]. It is used as a substrate material for graphene-based electronics and as a dielectric material in nanoelectronics devices. It finds application regarding corrosion resistance and antioxidation protective coatings [19]. Due to its high thermal conductivity and electrical insulation, h-BN is used in thermal management applications. h-BN is used in far-ultraviolet light-emitting devices. These applications leverage h-BN’s properties like high temperature stability, electrical insulation, and chemical resistance [18,20–21].

An interesting application of multilayer h-BN is its deposition on electrode surfaces as an electron blocking layer [22]. The electron tunneling through ultrathin h-BN crystalline barriers was investigated recently with experimental techniques showing exponential decay of the current with the number of layers [23]. Accurate density functional theory (DFT) calculations demonstrated the bandgap change in one to eight layers of h-BN with convergence at four layers to the bulk values [24].

Electron transport through two layers of graphene and h-BN was investigated with the non-equilibrium Green’s function (NEGF) method combined with DFT [25]. The results show similar tunneling currents for graphene and h-BN. This work rose questions on the ability of h-BN to effectively screen electron currents at the nanoscale. The electron transport at the nanometer scale differs significantly from the macroscopic transport, which makes the design of nanodevices a nontrivial problem. For macroscopic materials, the conductance, given via Ohm’s law and the current, is proportional to the conductor’s width and inversely proportional to the conductor’s length with a proportionality factor denoted as the specific conductivity of the material, directly related to the free path of the electrons. The free path in pristine graphene can be as long as 100 nm [26–27]. For nanoscale devices shorter than the free path of the electrons, Ohm’s law is not applicable, and the specific conductivity of the material is irrelevant to the transport properties. The conductance is provided by the Landauer formula of quantum conductance, with which the current through the device quantum levels is estimated [28].

In this work, we approach the quantum transport through metal/multilayer graphene/metal nanojunctions and compare it to the transport through metal/multilayer h-BN/metal nanojunctions. The choice of the metal electrode is platinum as platinum is often used in the field of electrochemistry and energy materials. In addition, we implement two possible defects in multilayer graphene, namely, Stone–Wales defects and graphitic nitrogen substitution. In the case of h-BN, we perform simulations with Stone–Wales defects. We investigate devices with A–B graphene stacking with one to six layers, which correspond to thicknesses of 0.5–3.0 nm, respectively. We provide comparison between the different junctions and explanation for the observed trends in the quantum electron transport. Our simulations are based on the NEGF method combined with linear combination of atomic orbitals (LCAO) DFT.

The study aims to provide practical insights into the minimal thickness of h-BN at which the tunneling currents diminish and the material could be used as an electron blocking layer in energy-related devices. The fundamental aspect of the study is to differentiate the electron transport mechanisms between graphene and h-BN at the nanoscale and compare them to their macroscopic properties. The role of several common defects on the electron transport will be elucidated, which is an important transition from pristine models to realistic applications.

## Methods

All calculations in this study are performed with NEGF combined with LCAO DFT as implemented in the QantumWise ATK software package [29]. We use the Perdew–Burke–Ernzerhof (PBE) functional [30] combined with the Fritz-Haber-Institut (FHI) pseudopotentials. A single-zeta polarized basis set was used for the Pt atoms and a double-zeta polarized basis set was used for all other atoms. Geometry optimization was performed with the LBFGS algorithm until all forces are converged below 0.05 eV·Å^−1^. The k-point density was selected to be four k-points per angstrom. In case of NEGF, the k-point density in the *c*-direction was selected to be 150 k-points per angstrom. Benchmark tests were performed using the NEGF method with Au electrodes instead of Pt, and the Heyd–Scuseria–Ernzerhof 2006 screened hybrid functional (HSE06) instead of PBE. The results obtained from those benchmark simulations showed slight numerical deviations; however, all trends were preserved.

The conductance through a nanoscale device is described by the Landauer formula shown in Equation 2,


[2] $$g=\frac{2e^{2}}{h}T,$$

where *g* is the conductance, *e* is the electron charge, *h* is Planck’s constant, and *T* is the transmission probability. The transmission probability can be calculated using Green’s function of the semi-infinite electrode–conductor–electrode system. The transmission probability is a function of the applied bias between the electrodes, *V*, and the energy of the electrons within the conductor, *E*; it is given by Equation 3:


[3] $$T\left(E,V\right)=\text{trace}\left[\Gamma_{\text{L}}G^{\text{R}}\Gamma_{\text{R}}G^{\text{A}}\right].$$

*G*^A^ and *G*^R^ are the advanced and the retarded Green’s function, respectively. Γ_L_ and Γ_R_ are the broadening functions of the electrodes. The Green’s function of the electrode–conductor–electrode system is given by Equation 4. *H*_C_ is the Hamiltonian matrix of the conductor, with *H*_L_ and *H*_R_ representing the Hamiltonian matrices of the left and the right electrode, respectively. *V*_CL_ and *V*_LC_ denote the interaction matrices between the left electrode and the channel. *V*_CR_ and *V*_RC_ denote the interaction matrices between the right electrode and the channel. *I*_L_, *I*_C_, and *I*_R_ denote the unity matrices.


[4] $$G={\left(\begin{array}{ccc} EI_{\text{L}}-H_{\text{L}} & -V_{\text{CL}} & 0\\ -V_{\text{LC}} & EI_{\text{C}}-H_{\text{C}} & -V_{\text{CR}}\\ 0 & -V_{\text{RC}} & HI_{\text{R}}-H_{\text{R}} \end{array}\right)}^{-1}.$$

The Hamiltonian matrices can be constructed using a different level of theories, depending on the complexity of the investigated system and the desired accuracy (i.e., tight binding approximation or DFT) [31]. The major computational problem of Equation 4 is the different dimensions of *H*_C_, *H*_L_, and *H*_R_. A series of linear algebra operations transforms Equation 4 to Equation 5, in which all matrices have finite dimensions:


[5] $$G_{\text{C}}\left(E\right)={\left(EI_{\text{C}}-H_{\text{C}}-\Sigma_{\text{L}}-\Sigma_{\text{R}}\right)}^{-1}.$$

Σ_L_ and Σ_R_ denote the self-energies of the left and the right electrode, respectively, given by Equation 6 and Equation 7:


[6] $$\Sigma_{\text{L}}=\left(-V_{\text{CL}}\right){\left(EI_{\text{L}}-H_{\text{L}}\right)}^{-1}\left(-V_{\text{LC}}\right),$$

[7] $$\Sigma_{\text{R}}=\left(-V_{\text{CR}}\right){\left(EI_{\text{R}}-H_{\text{R}}\right)}^{-1}\left(-V_{\text{RC}}\right).$$

The matrices *V*_CL_, *V*_LC_, *V*_CR_, and *V*_RC_ depend on the overlap of *H*_C_ with *H*_L_ and *H*_R_, respectively. *V*_CL_, *V*_LC_, *V*_CR_, and *V*_RC_ represent the escape rates of an electron from a conductance channel, for example, the probability of an electron to enter or leave the junction. The broadening functions Γ_L_ and Γ_R_ are derived from the self-energies of the electrodes and are given by Equation 8 and Equation 9:


[8] $$\Gamma_{\text{L}}=i\left[\Sigma_{\text{L}}-\Sigma_{\text{L}}^{†}\right],$$

[9] $$\Gamma_{\text{R}}=i\left[\Sigma_{\text{R}}-\Sigma_{\text{R}}^{†}\right].$$

The steady-state current through the device is given by Equation 10, where *e* is the electron charge, *f* is the Fermi function of the electrodes, μ_L_ and μ_R_ are the chemical potentials at the left and the right electrode, respectively, and *T*(*E*) is the transmission probability as a function of the electron energy. When the current is calculated for different applied biases, the current/voltage (*I*/*V*) curve is obtained.


[10] $$I=\frac{2e}{h}\int_{-\infty }^{+\infty }\text{d}ET\left(E\right)\left[f\left(E-\mu_{\text{L}}\right)-f\left(E-\mu_{\text{R}}\right)\right]$$

## Results and Discussion

We start our calculations with a comparison of the electronic properties of graphite, bulk h-BN, graphite with Stone–Wales defect, graphitic nitrogen-doped graphite, and bulk h-BN with Stone–Wales defect. For graphite and bulk h-BN, we adopt a graphite unit cell with A–B stacking consisting of two layers and two atoms per layer. We perform geometry optimization, density of states (DOS) calculations, and band structure calculations. For graphite and h-BN with Stone–Wales defects, we use a 4 × 4 × 1 supercell. The Stone–Wales defect is located in one of the layers, while the second layer is kept defect-free in order to reduce the defect concentration. The Stone–Wales defect is obtained by rotation of a C–C bond or a B–N bond by 90°. The geometry is optimized, and DOS and band structure are calculated. The graphitic nitrogen doping is performed for a 4 × 4 × 1 supercell with one nitrogen atom substituting one carbon atom per layer.

**Figure 1:**
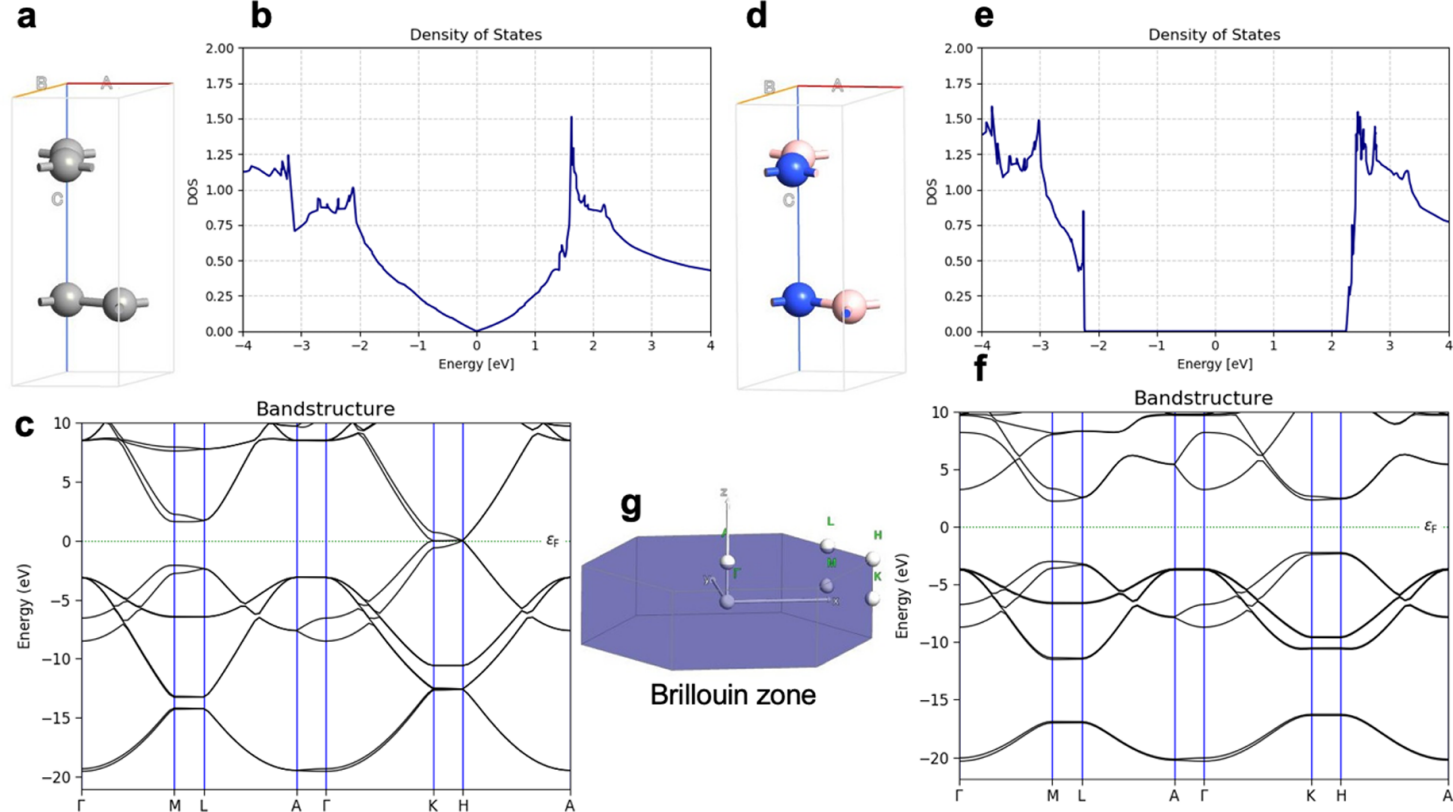
Modeling of graphite and h-BN. (a) Unit cell of graphite, (b) DOS of graphite, (c) band structure of graphite, (d) unit cell of h-BN, (e) DOS of h-BN, (f) band structure of h-BN, and (g) schematic of the Brillouin zone.

Figure 1 shows the unit cell of graphite, the DOS of graphite, the band structure of graphite, the unit cell of h-BN, the DOS of h-BN, the band structure of h-BN, and a schematic of the Brillouin zone. Graphite is a zero-bandgap semiconductor, while h-BN is a large-bandgap semiconductor with a direct bandgap of 4.58 eV and an indirect bandgap of 4.46 eV. The experimental optical bandgap of h-BN was measured to be approximately 6.0 eV [32]. The difference between the computed bandgap in our work and the experimental results can be attributed to the well-known underestimation of the bandgap by pure DFT methods [30].

**Figure 2:**
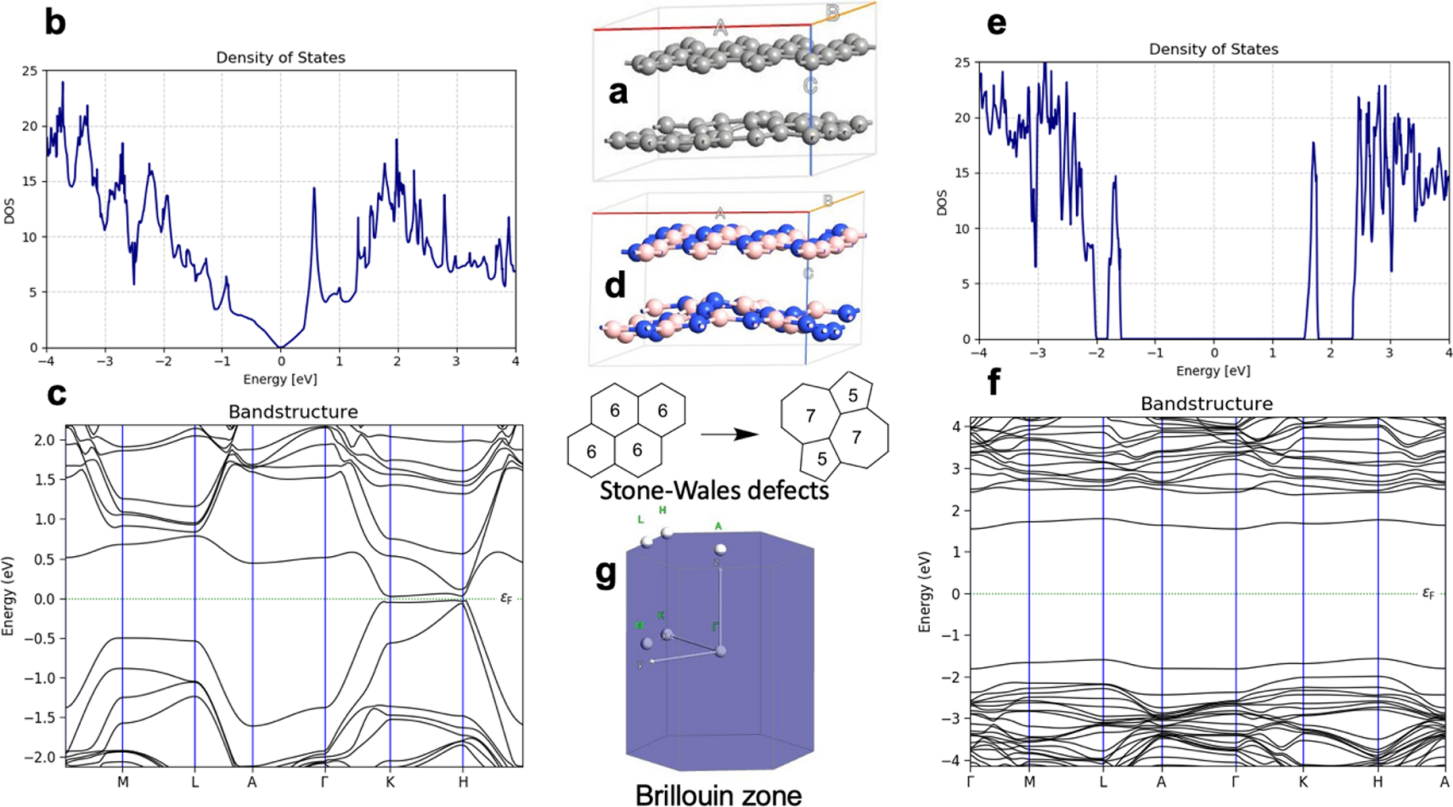
Modeling of graphite and h-BN with Stone–Wales defect. (a) Unit cell of defective graphite, (b) DOS of defective graphite, (c) band structure of defective graphite, (d) unit cell of defective h-BN, (e) DOS of defective h-BN, (f) band structure of defective h-BN, and (g) schematic of the Brillouin zone.

**Figure 3:**
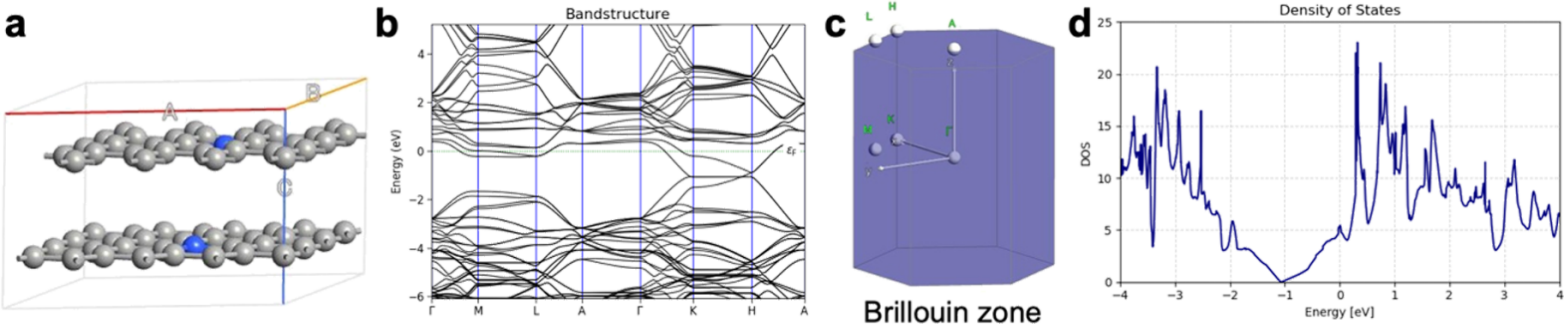
Modeling of N-doped graphite. (a) Unit cell of N-doped graphite, (b) band structure of N-doped graphite, (c) schematic of the Brillouin zone, and (d) DOS of N-doped graphite.

Figure 2 shows properties of graphite and h-BN with Stone–Wales defects, specifically, the unit cell of defective graphite, the DOS of defective graphite, the band structure of defective graphite, the unit cell of defective h-BN, the DOS of defective h-BN, the band structure of defective h-BN, and a schematic of the Brillouin zone. Stone–Wales defects were inserted in every second layer in the materials. In graphite, the Stone–Wales defect opens a direct bandgap of 0.06 eV and an indirect bandgap of 0.05 eV. In h-BN, the Stone–Wales defect leads to defect levels within the bandgap and narrows the direct gap to 3.31 eV and the indirect gap to 3.11 eV, which is in line with reported theoretical results [33]. Figure 3 shows the unit cell of N-doped graphite, the band structure of N-doped graphite, a schematic of the Brillouin zone, and the DOS of N-doped graphite. The electronic properties show metallic character, which was previously reported for single layer graphene with graphitic nitrogen doping [7].

**Figure 4:**
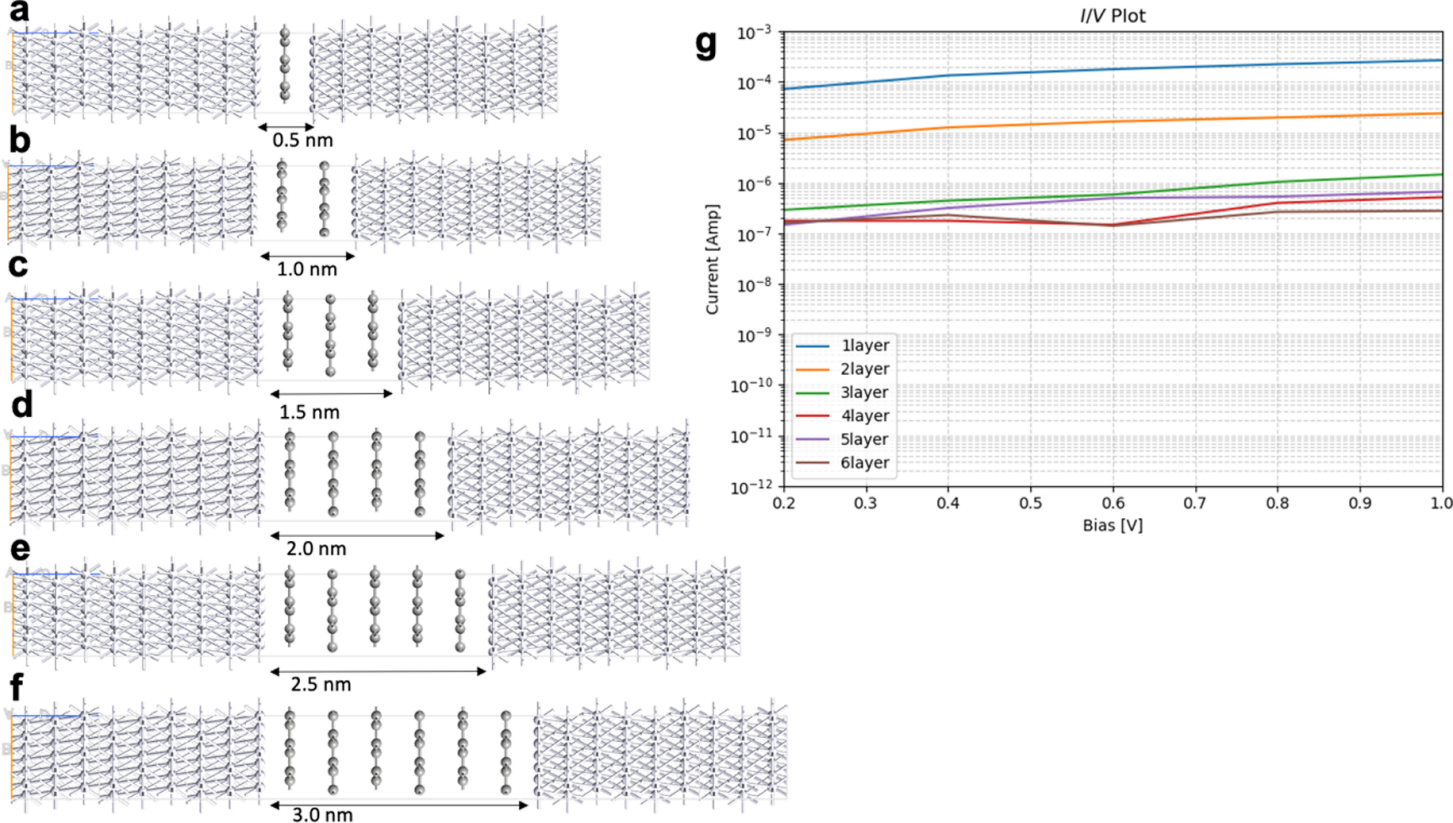
Geometry of Pt/graphene/Pt junctions with (a) one layer, (b) two layers, (c) three layers, (d) four layers; (e) five layers, and (f) six layers; (g) current/voltage (*I*/*V*) plot in logarithmic scale.

**Figure 5:**
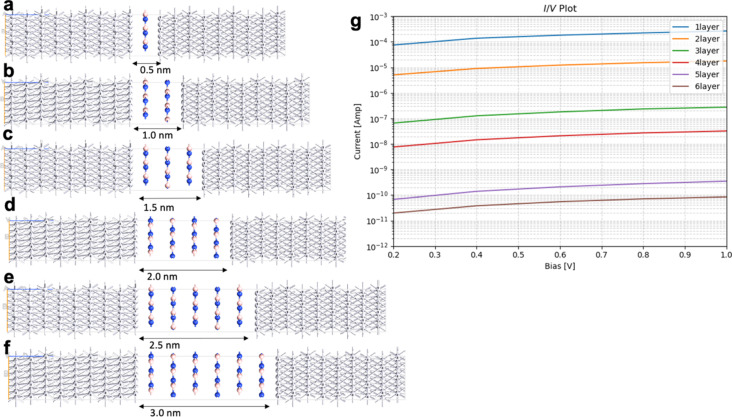
Geometry of Pt/h-BN/Pt junctions with (a) one layer, (b) two layers, (c) three layers, (d) four layers, (e) five layers, (f) six layers; (g) current/voltage (*I*/*V*) plot in logarithmic scale.

The results for the electron transport through graphene with one to six layers, compared to h-BN with one to six layers, are summarized in Figure 4, Table 1, Figure 5, and Table 2. Those junctions correspond to 0.5–3.0 nm thickness. The junctions in Figure 4 and Figure 5 are prepared from optimized unit cells of Pt(111) surface, graphene, and h-BH using the QuantumWise ATK interface builder. The process involves strain minimization in the lattice mismatch by symmetry multiplication of the unit cells. The junctions shown in Figure 4 and Figure 5 have 1.06% distributed strain over Pt and graphene or h-BN. The distance between graphene or h-BN and the Pt surface is set to 3.2 Å. The central region includes six layers of the source electrode and six layers of the drain electrode. The electrode supercells are 3 × 3 × 3 Pt unit cells. The (*I*/*V*) curves are computed using the methodology described in Equations 2–10. The current is computed for biases in range between 0.0 and 1.0 V with steps of 0.2 V.

**Table 1:** Calculated currents in amperes for Pt/graphene/Pt junction with one to six layers.

bias [V]	one layer	two layers	three layers	four layers	five layers	six layers

0.2	7.25·10^−5^	7.10·10^−6^	2.93·10^−7^	1.78·10^−7^	1.48·10^−7^	1.63·10^−7^
0.4	1.35·10^−4^	1.26·10^−5^	4.46·10^−7^	1.78·10^−7^	3.21·10^−7^	2.31·10^−7^
0.6	1.79·10^−4^	1.65·10^−5^	5.88·10^−7^	1.49·10^−7^	5.02·10^−7^	1.42·10^−7^
0.8	2.25·10^−4^	1.98·10^−5^	1.05·10^−6^	4.03·10^−7^	5.35·10^−7^	2.68·10^−7^
1.0	2.69·10^−4^	2.39·10^−5^	1.47·10^−6^	5.22·10^−7^	6.72·10^−7^	2.80·10^−7^

**Table 2:** Calculated currents in amperes for Pt/h-BN/Pt junction with one to six layers.

bias [V]	one layer	two layers	three layers	four layers	five layers	six layers

0.2	7.56 10^−5^	5.09·10^−6^	6.59·10^−8^	7.61·10^−9^	6.73·10^−11^	1.97·10^−11^
0.4	1.39·10^−4^	9.10·10^−6^	1.27·10^−7^	1.46·10^−8^	1.40·10^−10^	3.79·10^−11^
0.6	1.83·10^−4^	1.23·10^−5^	1.81·10^−7^	2.10·10^−8^	2.10·10^−10^	5.49·10^−11^
0.8	2.25·10^−4^	1.53·10^−5^	2.36·10^−7^	2.72·10^−8^	2.80·10^−10^	7.08·10^−11^
1.0	2.66·10^−4^	1.78·10^−5^	2.77·10^−7^	3.24·10^−8^	3.50·10^−10^	8.46·10^−11^

The computed current for one layer in the Pt/graphene/Pt junction and one layer in the Pt/h-BN/Pt junction is 2.6·10^−4^ A for 1.0 V of applied bias. The results are identical and, for one layer, the current flow through graphene and h-BN is indistinguishable. There is no evidence that graphene is more conductive than h-BN perpendicular to the planes at 0.5 nm thickness despite the profound difference in electronic properties such as the bandgap (Figure 1). The results are in line with previous calculations with similar methods [25]. The computed currents for two layers in the Pt/graphene/Pt junction and two layers in the Pt/h-BN/Pt junction are ca. 10^−5^ A for 1.0 V of applied bias. As the thickness increases to three and four layers, the computed currents for graphene drop to ca. 10^−6^ and ca. 10^−7^ A, respectively, while the computed currents for h-BN drop an order of magnitude lower to ca. 10^−8^ A. At a thickness of 2 nm, the difference in electron transport through graphene and h-BN is significant. As we increase the thickness to five and six layers, the computed currents for the graphene junction remain similar to those of four layers and converge to a value of ca. 10^−7^ A, while the currents through the h-BN junction drop to 10^−10^ and 10^−11^ A, respectively. At 3 nm thickness, the difference of the conductance between graphene and h-BN is four orders of magnitude, clearly demonstrating the conducting nature of multilayer graphene and the electron-current isolating nature of multilayer h-BN. The current through the graphene junction drops exponentially from one to four layers and then converges to a linear dependence for four, five, and six layers. The current through h-BN drops exponentially in the range from one to six layers. Our computational results for electron transport through two-layer and four-layer h-BN junctions match the experimentally reported trends [23]. The experimental study by Britnell et al. [23] investigated electron tunneling through ultrathin crystalline layers of h-BN sandwiched between conductive materials such as graphene, graphite, and gold. The authors fabricated vertical tunnel junctions with h-BN barriers ranging from one to four atomic layers and demonstrated that the tunneling current decreases exponentially with barrier thickness, confirming quantum mechanical tunneling behavior. Current–voltage characteristics showed linear dependence at low bias and exponential growth at higher voltages. Conductive atomic force microscopy measurements revealed highly uniform, defect-free tunneling across atomically flat h-BN terraces, with breakdown fields near 1 GV·m^−1^. These findings establish h-BN as a high-quality, atomically thin dielectric suitable for next-generation tunneling devices and graphene-based heterostructures. From the results in Figure 4, Figure 5, Table 1, and Table 2 we can conclude that, at thicknesses of one to three layers, the transport is dominated by tunneling currents and is not related to the electronic properties of the materials. For four to six layers of thickness, the transport mechanisms differ between graphene and h-BN, where we have ballistic transport through quantum levels in graphene with linear dependence on the thickness and tunneling currents through h-BN with exponential dependence on the thickness. Those conclusions are supported by the analysis of the transmission spectra at 0.0 and 0.8 V bias for graphene and h-BN junctions, shown in Figure S1 and Figure S2 of Supporting Information File 1.

An interesting difference between the current/voltage plots in Figure 4g and Figure 5g is the slight drop of current with applied bias for the graphene junctions with five and six layers, which is missing for h-BN junctions. We assign this result to the phenomenon of negative differential resistance (NDR), in which the electric current through a device decreases as the applied voltage increases within a certain voltage range; this results in a region of the current–voltage curve where the differential conductance (d*I*/d*V*) is negative. This counterintuitive behavior contrasts with ordinary resistive materials, where current always rises with voltage. NDR occurs in various electronic systems, such as tunnel diodes, resonant tunneling diodes, and molecular junctions, and arises from quantum mechanical effects like tunneling or charge carrier trapping. In short molecular and nanoscale junctions, NDR is assigned to localization of a conducting level close to one of the electrodes because of the applied bias. The localized level reduces the electron transport. The effect in our work is observed only in longer graphene junctions as the conductance is achieved through quantum levels in the bias window as shown in below Figure 7 and in Supporting Information File 1. Longer junctions favor better localization of the levels. In contrast, the conductance in h-BN is based on tunneling currents; thus, NDR is not observed and not expected.

**Figure 6:**
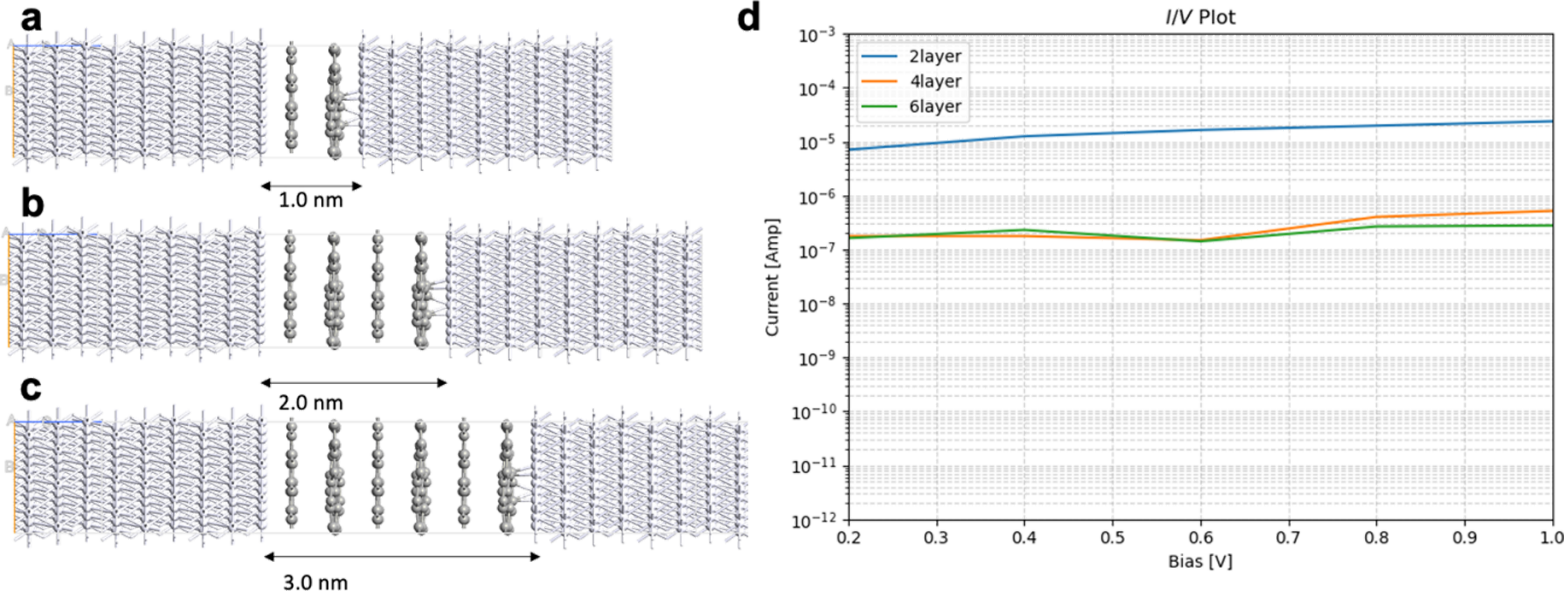
Geometry of Pt/graphene with Stone–Wales defect/Pt junctions with (a) two layers, (b) four layers, and (c) six layers; (d) current/voltage (*I*/*V*) plot in logarithmic scale.

Graphene lattices often exhibit structural and electronic defect states leading to deviations from the electronic properties of pristine graphene [7]. Here, we consider the effect of the Stone–Wales defect; this is a result of thermal structural isomerization of the graphene lattice where a bond is rotated by 90°, resulting to the isomerization of a 6–6 ring structure to an isoelectronic 7–5 ring structure. The Stone–Wales defect does not influence the work function of graphene. It opens a bandgap in the electronic structure. The data in Table 3 and the compared results of graphene junctions without (Table 1 and Figure 4) and with Stone–Wales defect (Figure 6) show that, at higher applied biases (1.0 V), all junctions have one order of magnitude higher current through graphene with Stone–Wales defect compared to pristine graphene junctions with the same thickness. The results are counterintuitive as the open bandgap should reduce the conductance. To understand this result, we compute the binding energy of pristine graphene and graphene with Stone–Wales defect to the Pt(111) surface. In those calculations, we use two graphene layers with a 4 × 4 × 1 supercell. The Stone–Wales defect is in the layer facing the Pt surface. Pt is modeled by six layers where the bottom three layers have the atomic positions fixed and the top three layers are fully relaxed. To the top of the slab are added 10 Å vacuum. The binding energy between graphene (and graphene with Stone–Wales defect) and the Pt(111) surface is calculated as the difference between the energy of the entire slab and the energies of graphene and the Pt(111) surface subtracted from it. The binding energy of pristine graphene to the Pt(111) surface is 1.18 eV, and the binding energy of the graphene with Stone–Wales defect to the Pt surface is 1.85 eV. Thus, the Stone–Wales defect leads to stronger binding to the metal surface. Such results were reported using experimental and theoretical studies for the interaction of azupyrene and pyrene with a Cu surface [16]. The metal electrode/graphene binding has strong influence on the electron transport properties. In Equations 6 and 7, the self-energies of the electrodes depend on the interaction matrices denoted with *V*_CL_, *V*_LC_, *V*_CR_, and *V*_RC_. Stronger binding leads to larger values of *V*_CL_, *V*_LC_, *V*_CR_, and *V*_RC_, and to larger values of the self-energies. The self-energies determine the broadening functions in Equations 8 and 9, and the broadening functions influence the transmission probability in Equation 3. Larger transmission probability leads to larger currents in Equation 10. Thus, the enhanced electrode/graphene interaction due to the Stone–Wales defects leads to enhanced current through the device.

**Table 3:** Calculated currents in amperes for Pt/graphene with Stone–Wales defect/Pt junctions with two layers, four layers, and six layers.

bias [V]	two layers	four layers	six layers

0.2	1.74·10^−5^	2.82·10^−7^	1.48·10^−7^
0.4	3.35·10^−5^	8.81·10^−7^	4.59·10^−7^
0.6	4.23·10^−5^	1.43·10^−6^	9.69·10^−7^
0.8	5.02·10^−5^	2.21·10^−6^	1.16·10^−6^
1.0	6.02·10^−5^	3.73·10^−6^	1.47·10^−6^

**Figure 7:**
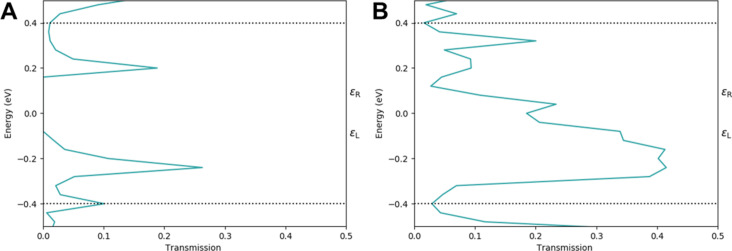
Transmission spectra of (a) Pt/graphene/Pt and (b) Pt/graphene with Stone–Wales defect/Pt junctions with six layers and 0.8 V applied bias.

The conclusions are supported by analysis of the transmission spectra of Pt/graphene/Pt and Pt/graphene with Stone–Wales defect/Pt junctions with six layers, which are shown in Figure 7A and Figure 7B, respectively. The transmission spectra are plotted for an applied bias of 0.8 V. The bias window is denoted with dashed lines. Figure 7A shows two distinct, sharp, resonance peaks in the bias window at −0.2 and 0.2 eV. Sharp transmission peaks correspond to localized quantum states. The spectrum in Figure 7B is characterized by one broad peak with high intensity at −0.2 eV and a sharp peak with lower intensity at 0.2 eV. Broadened peaks correspond to delocalized quantum states with good electron transmission and high amplitudes at both electrodes.

**Figure 8:**
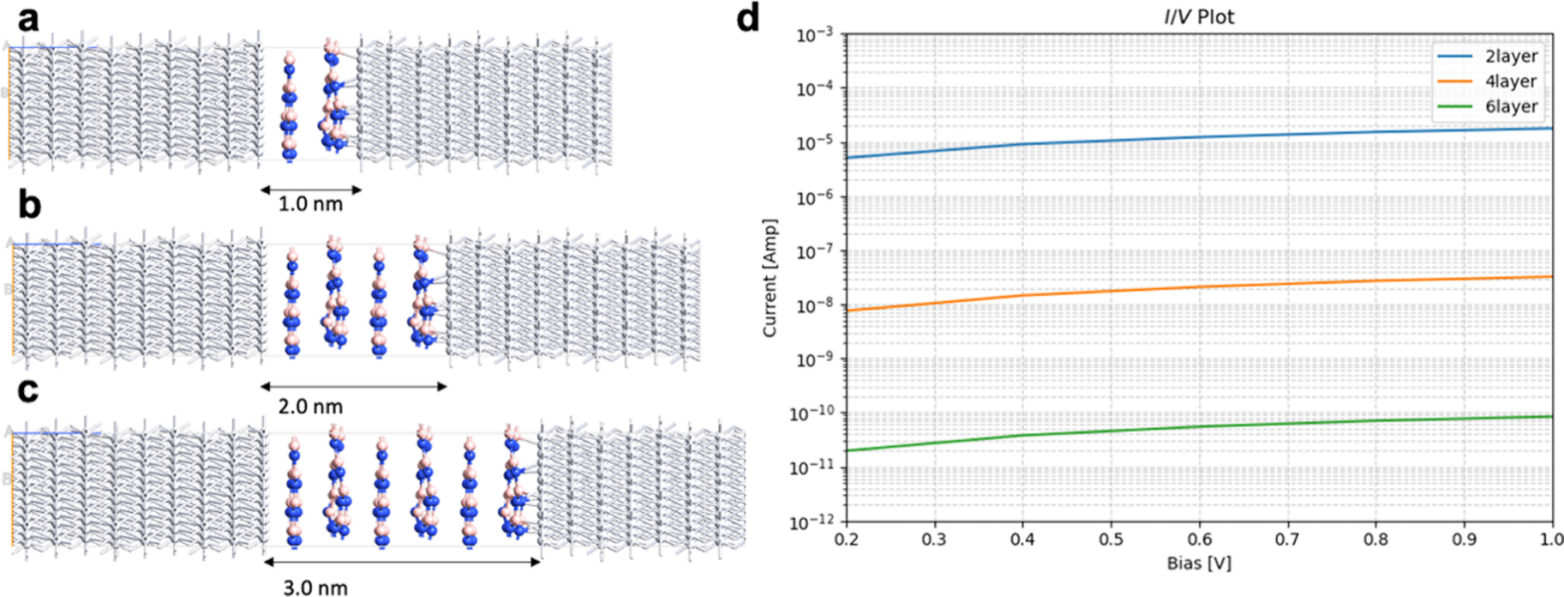
Geometry of Pt/h-BN with Stone–Wales defect/Pt junctions with (a) two layers, (b) four layers, and (c) six layers; (d) current/voltage (*I*/*V*) plot in logarithmic scale.

Figure 8 provides results for the electron transport through the Pt/h-BN with Stone–Wales defect/Pt junctions. The computed currents for two layers, four layers, and six layers Pt/h-BN with Stone–Wales defect/Pt junctions in the bias range of 0.0 to 1.0 V are summarized in Table 4. Similar to graphene with and without Stone–Wales defect, h-BN with Stone–Wales defect shows higher currents than the defect-free junctions. However, the difference between the currents through the h-BN junctions and the currents through h-BN with Stone–Wales defect is only approximately 20%, unlike the one order of magnitude in the case of graphene. Our understanding is that, in the h-BN junctions with and without Stone–Wales defect, the dominant transport mechanism is tunneling, which is not affected significantly by interaction of the h-BN with Stone–Wales defect with the Pt electrode nor by the self-energies of the electrodes in Equations 6 and 7. Thus, the Stone–Wales defect enhances the electron transport, however, to a lesser extend compared to the graphene junctions.

**Table 4:** Calculated currents in amperes for Pt/h-BN with Stone–Wales defect/Pt junctions with two layers, four layers, and six layers.

bias [V]	two layers	four layers	six layers

0.2	9.68·10^−6^	1.22·10^−8^	2.26·10^−11^
0.4	1.72·10^−5^	2.32·10^−8^	4.47·10^−11^
0.6	2.32·10^−5^	3.34·10^−8^	6.60·10^−11^
0.8	2.91·10^−5^	4.36·10^−8^	8.78·10^−11^
1.0	3.45·10^−5^	5.38·10^−8^	1.10·10^−10^

**Figure 9:**
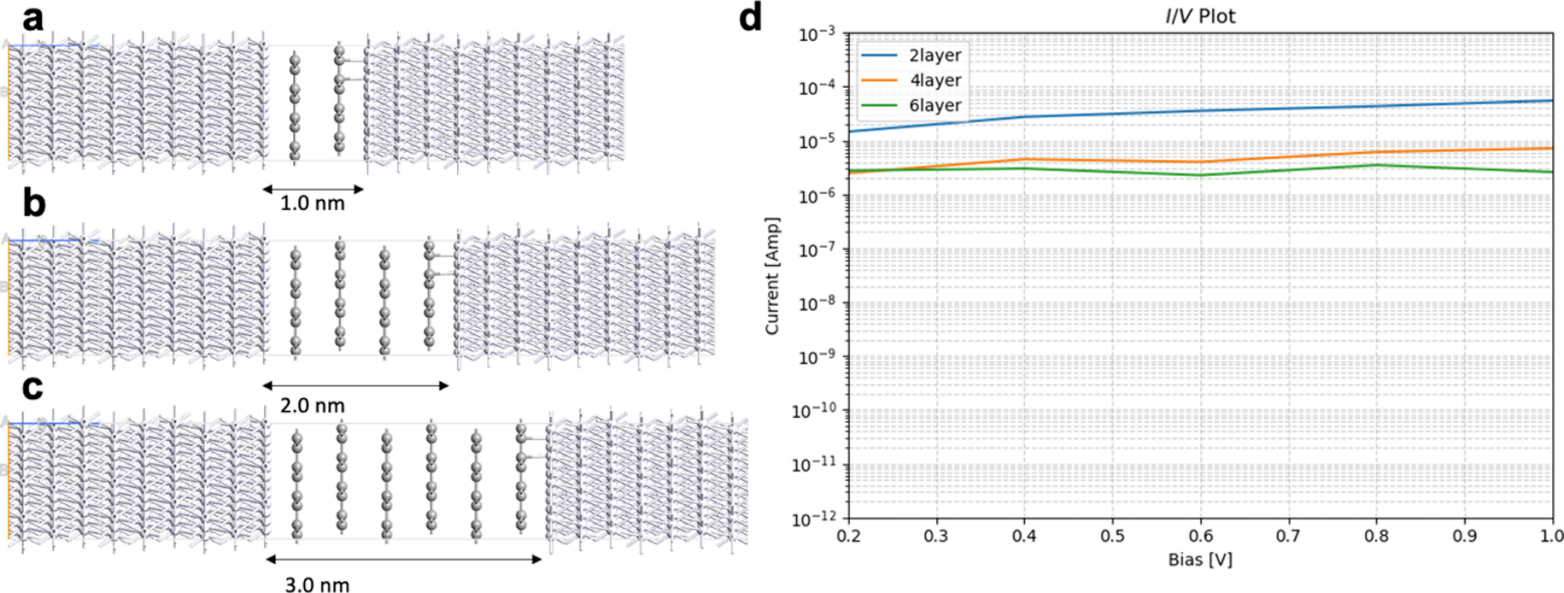
Geometry of Pt/graphene with N-doping/Pt junctions with (a) two layers, (b) four layers, and (c) six layers; (d) current/voltage (*I*/*V*) plot in logarithmic scale.

Figure 9 compares the electron transport through Pt/graphene with N-doping/Pt junctions. N-doped graphene shows greater values of the computed currents for all junctions (two layers, four layers, and six layers) compared to pristine graphene (Figure 4). The results are summarized in Table 5. We computed the binding energy of N-doped graphene to the Pt(111) surface. In these calculations, we use two N-doped graphene layers with 4 × 4 × 1 supercells. Pt is modeled by six layers where the bottom three layers have the atomic positions fixed and the top three layers are fully relaxed. To the top of the slab are added 10 Å vacuum. The binding energy between the N-doped graphene and Pt(111) surface is calculated as the difference between the energy of the entire slab and the energies of graphene and the Pt(111) surface subtracted from it. The binding energy of the N-doped graphene to the Pt(111) surface is 1.82 eV. The enhanced binding between the defective graphene and the metal electrodes is believed to be the reason for the improved transport properties. The current through the N-doped graphene junctions is one order of magnitude greater for four-layer and six-layer junctions.

**Table 5:** Calculated currents in amperes for Pt/graphene with N doping/Pt junction with two layers, four layers, and six layers.

bias [V]	two layers	four layers	six layers

0.2	1.47·10^−5^	2.49·10^−6^	2.78·10^−6^
0.4	2.76·10^−5^	4.51·10^−6^	3.03·10^−6^
0.6	3.58·10^−5^	4.04·10^−6^	2.28·10^−6^
0.8	4.37·10^−5^	6.15·10^−6^	3.51·10^−6^
1.0	5.54·10^−5^	7.21·10^−6^	2.61·10^−6^

## Conclusion

In this work, we investigated the electron transport perpendicular to the planes between Pt electrodes through multilayer graphene and h-BN junctions, as well as graphene junctions with Stone–Wales defect and N-doping, and h-BN with Stone–Wales defect. We employed the NEGF method combined with LCAO DFT. We also investigated the thickness dependence of the current by performing simulations of junctions with one to six layers, corresponding, respectively, to 0.5–3.0 nm. Our simulations show different regimes of the current for graphene and h-BN. The electron transport through multilayer graphene nanojunctions is ballistic, converging with the thickness to ca. 10^−7^ A. The current through h-BN exhibits tunneling behavior, dropping exponentially as the thickness of the layers is increased. For one-, two-, and three-layer junctions, the current through graphene and h-BN junctions is similar as the tunneling currents are dominant. For six-layer junctions, the current through graphene is four orders of magnitude higher than through h-BN, demonstrating the electron blocking properties of 3.0 nm thick h-BN layers. In addition, we investigated the currents through two-, four-, and six-layer junctions of graphene and h-BN with Stone–Wales defects. In both cases, the currents through the defective junctions were higher than the currents though the pristine junctions. We concluded that the enhanced electron transport properties are the result of stronger binding interaction of defective graphene and h-BN to the metal electrodes. The stronger binging to the electrodes enhances the self-energies of the electrodes and, as a result, maximizes the transmission probability. Thus, the defects increase the current through the nanoscale junctions.

## Supporting Information

File 1 Transmission spectra for 0.0 and 0.8 V applied biases for all investigated junctions.

## Data Availability

Data generated and analyzed during this study is available from the corresponding author upon reasonable request.
