# Supplementary material for: Electron transport through nanoscale multilayer graphene and hexagonal boron nitride junctions
Source: Beilstein J Nanotechnol. 2025 Nov 24;16:2132–43. doi: 10.3762/bjnano.16.147 (PMC12667729; doi:10.3762/bjnano.16.147)
Supplement: File 1 — Transmission spectra for 0.0 and 0.8 V applied biases for all investigated junctions. [file Beilstein_J_Nanotechnol-16-2132-s001.pdf]

BEILSTEIN JOURNAL OF NANOTECHNOLOGY

Supporting Information

for

Electron transport through nanoscale multilayer graphene and hexagonal boron nitride junctions

Aleksandar Staykov and Takaya Fujisaki

Beilstein J. Nanotechnol. **2025,** *16,* 2132–2143. doi:10.3762/bjnano.16.147

Transmission spectra for 0.0 and 0.8 V applied biases for all investigated junctions

License and Terms: This is a supporting information file under the terms of the Creative Commons Attribution License (https://creativecommons.org/licenses/by/4.0). Please note that the reuse, redistribution and reproduction in particular requires that the author(s) and source are credited and that individual graphics may be subject to special legal provisions.
The license is subject to the *Beilstein Journal of Nanotechnology* terms and conditions: (https://www.beilstein-journals.org/bjnano/terms)

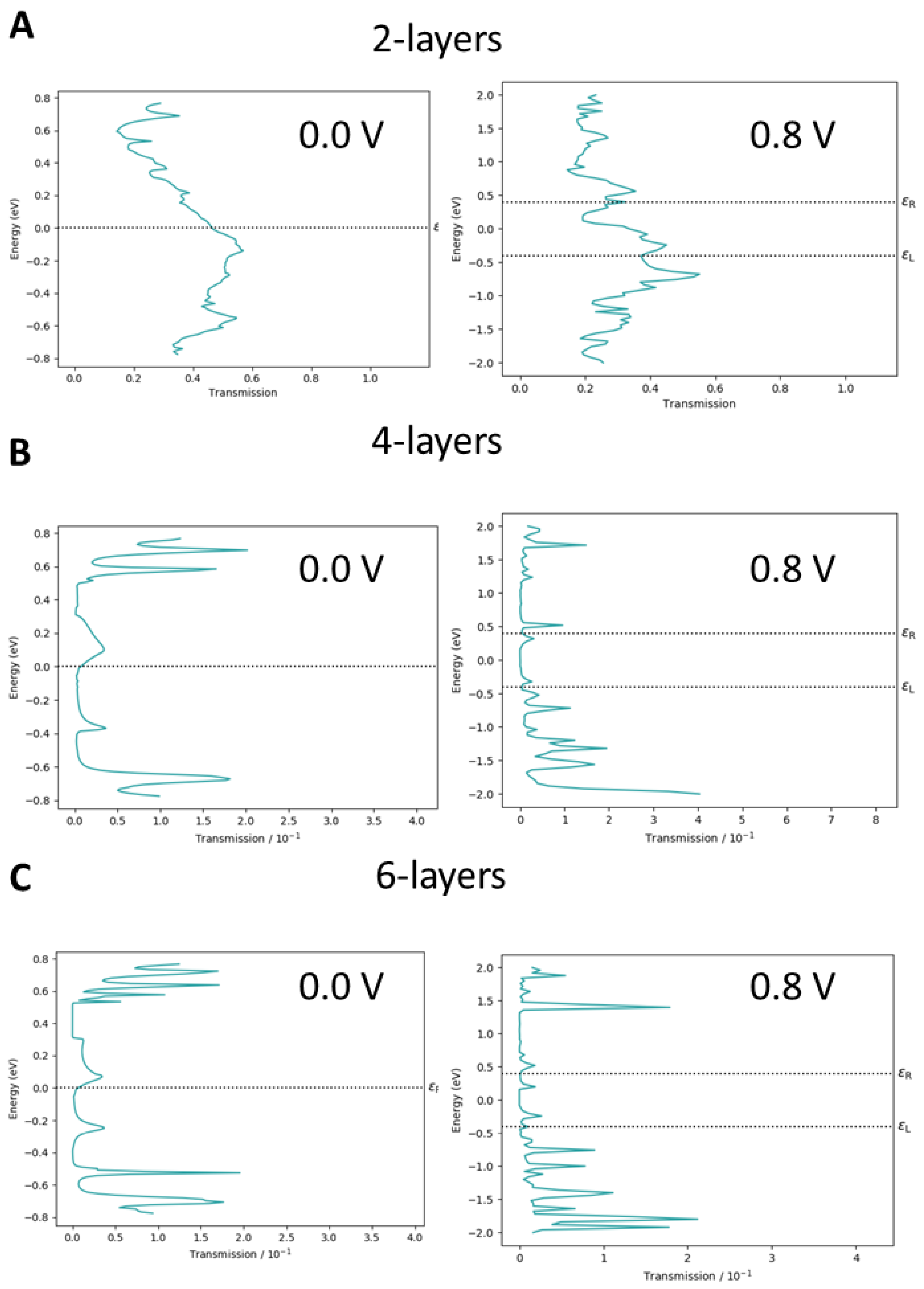

Figure S1: Transmission spectra for 0.0 and 0.8 V applied biases through Pt/graphene/Pt junctions with (A) two layers, (B) four layers, and (C) six layers.

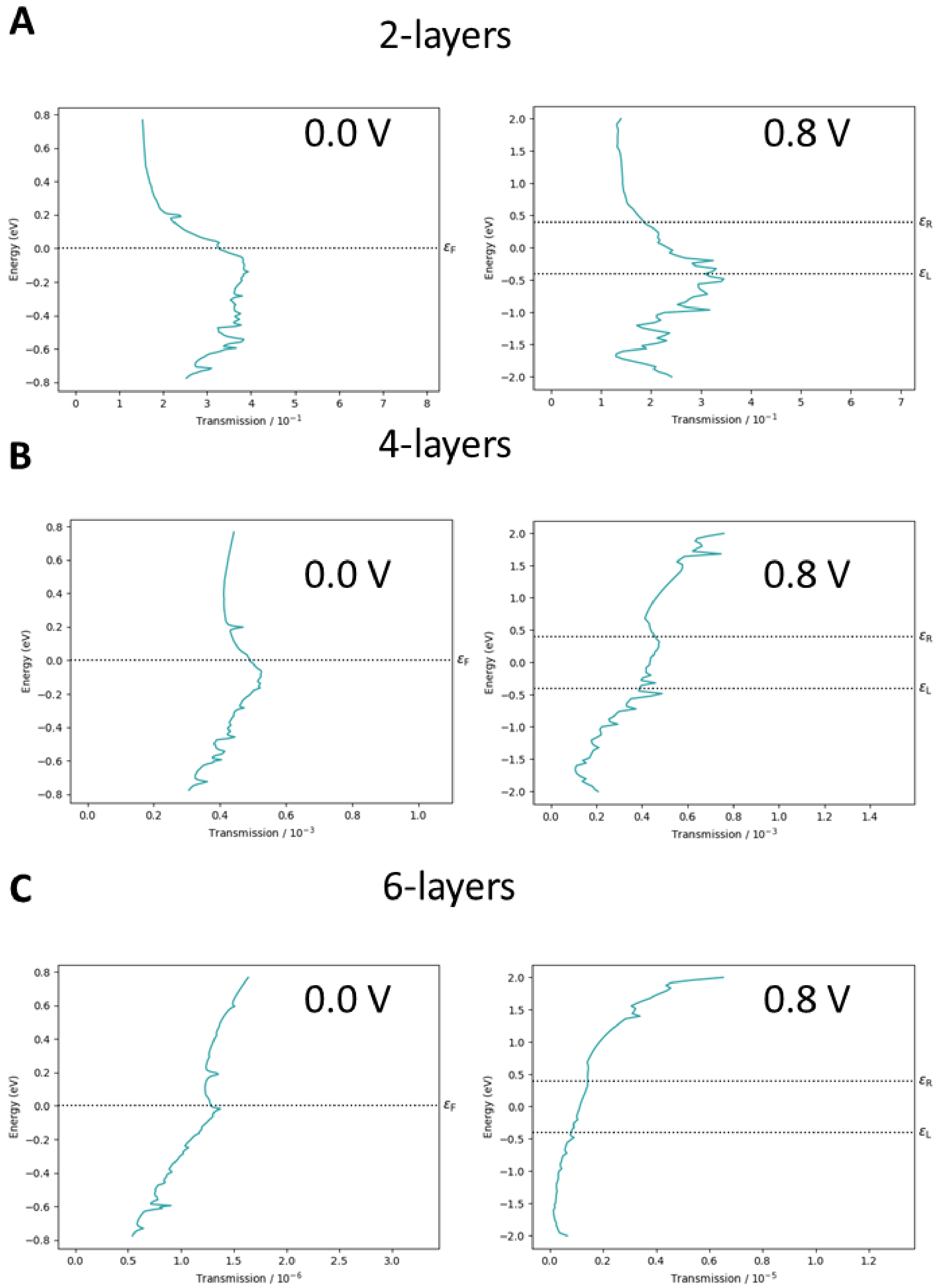

Figure S2: Transmission spectra for 0.0 and 0.8 V applied biases through Pt/h-BN/Pt junctions with (A) two layers, (B) four layers, and (C) six layers.

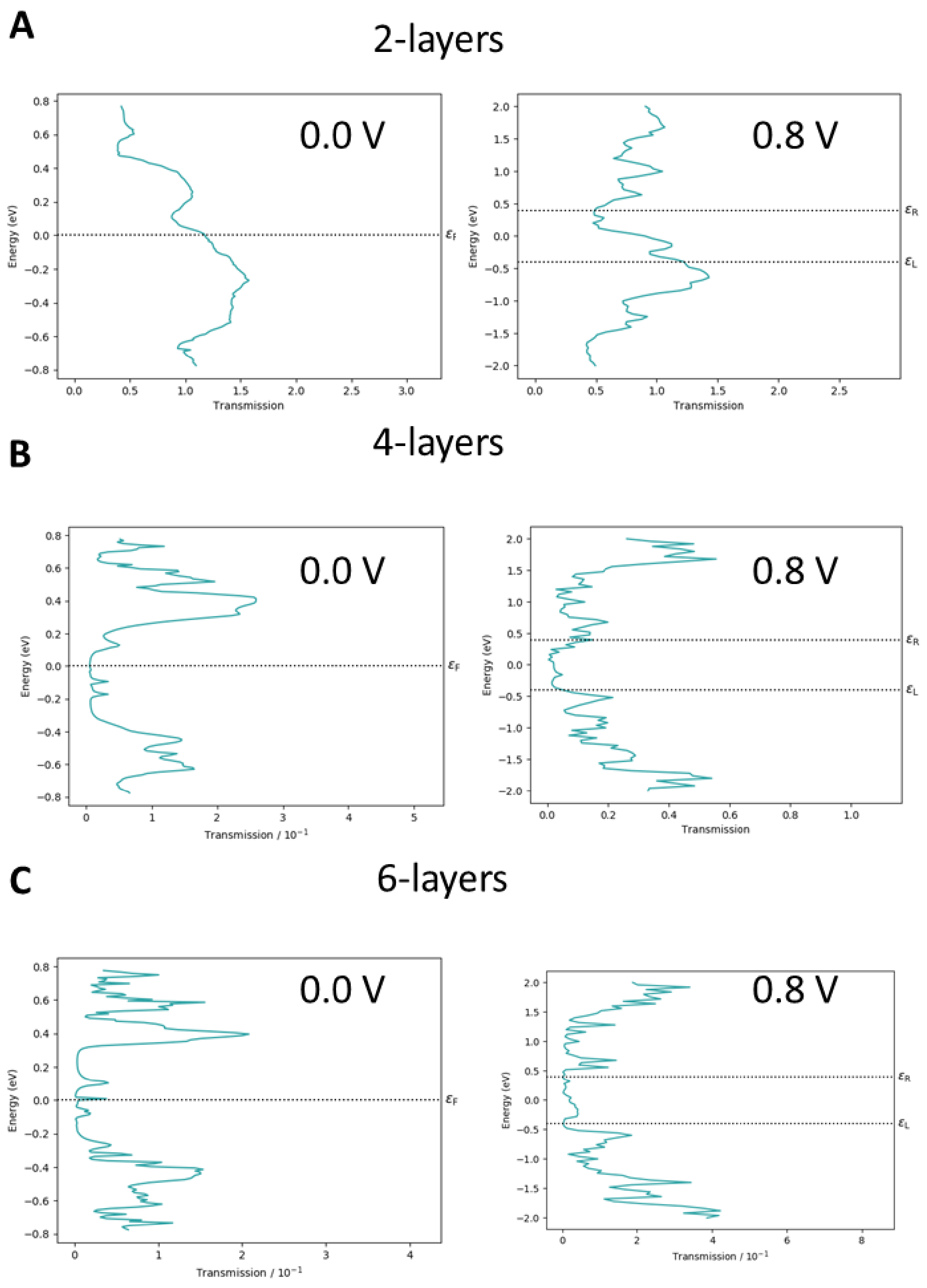

Figure S3: Transmission spectra for 0.0 and 0.8 V applied biases through Pt/graphene with Stone–Wales defect/Pt junction with (A) two layers, (B) four layers, and (C) six layers.

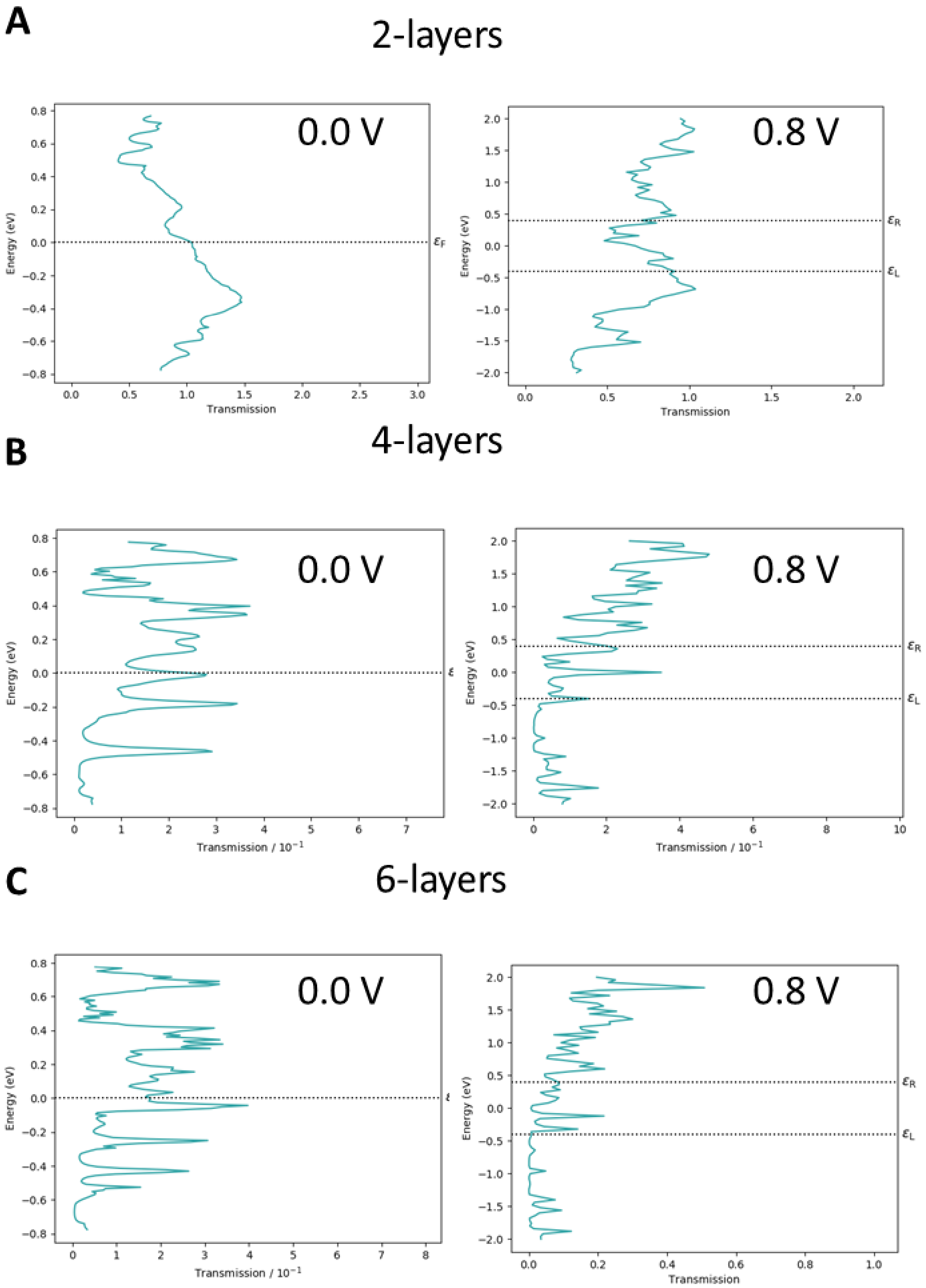

Figure S4: Transmission spectra for 0.0 and 0.8 V applied biases through Pt/graphene with N-doping/Pt junction with (A) two layers, (B) four layers, and (C) six layers.

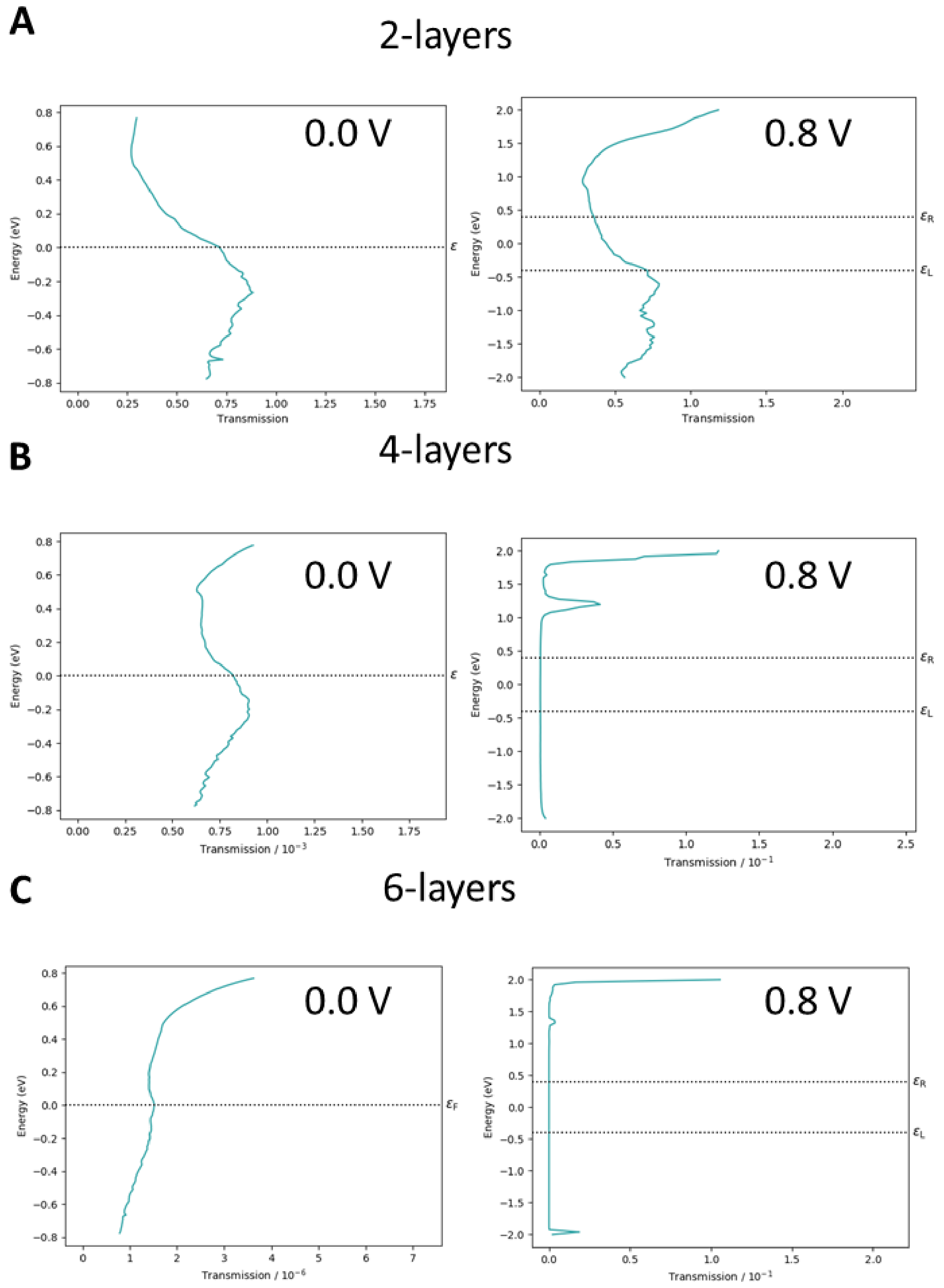

Figure S5: Transmission spectra for 0.0 and 0.8 V applied biases through Pt/h-BN with Stone–Wales defect/Pt junction with (A) two layers, (B) four layers, and (C) six layers.
